# Successful treatment of a patent urachus concurrent with pyocele in a newborn: A case report

**DOI:** 10.1097/MD.0000000000029187

**Published:** 2022-05-27

**Authors:** Susie Kang, Erika Imura, Yoo-Jin Kim, Shin Ae Yoon, Ji Hyuk Lee, Jin-Woo Park

**Affiliations:** a Department of Pediatrics, Chungbuk National University Hospital, Chungbuk National University College of Medicine, Cheongju, Korea; b Department of Surgery, Chungbuk National University Hospital, Chungbuk National University College of Medicine, Cheongju, Korea.

**Keywords:** fistulography, newborn, patent urachus, pyocele, umbilical cord cyst

## Abstract

**Rationale:**

A patent urachus is a rare congenital anomaly that atypically presents as an umbilical cord cyst or large umbilical cord. Here we describe a case of a giant umbilical cord cyst in a newborn diagnosed as a patent urachus.

**Patient concerns:**

A male infant with a birth weight of 3260 g was transferred because of an antenatally diagnosed giant umbilical cord cyst accompanied by yellowish discharge and granulation in the umbilical cord after birth.

**Diagnoses:**

Patent urachus

**Intervensions:**

The patent urachus was treated by excision of the urachal remnant followed by partial cystectomy.

**Outcomes:**

Postoperative orchitis with pyocele occurred and was treated with a course of antimicrobial therapy; and no other complications developed.

**Lessons:**

Newborns with a giant umbilical cord or umbilical cord cysts should be examined for possible accompanying urachal anomalies, even if antenatal ultrasound shows no other suspicious findings, to prevent delayed diagnosis and subsequent complications.

## 1 Introduction

Urachal remnants are rare developmental anomalies that occur because of defects in the developmental process during the fetal period. During normal development, the proximal part of the allantois extends into the urogenital sinus, and the remaining part of the allantois is surrounded by the umbilical cord, within which it projects outwards to form a fibrous connection between the apex of the bladder and the umbilicus called the urachus.^[1]^ This fibrous tube-like structure should be filled and closed off by the 12th week of development to become the median umbilical ligament. However, incomplete obliteration of the urachus leads to a spectrum of urachal anomalies, depending on the location and extent of incomplete obliteration. Newborns with urachal remnants usually present with symptoms of near-persistent umbilical leakage of urine, inflammation (e.g., omphalitis, cellulitis), irritability, a giant umbilical cord, and a palpable abdominal mass upon birth or soon after.^[2,3]^

Urachal anomalies are usually diagnosed through antenatal ultrasound, but the appearance of the above-mentioned symptoms or abnormal physical findings suggesting urachal remnants should be evaluated to diagnose and clarify the type of urachal anomalies.^[3]^ These abnormalities can present as follows: A patent urachus is a urachal remnant with an intact hollow lumen connecting the bladder to the umbilicus; a urachal sinus has a hollow, open pocket at the umbilical end of the urachus with a partially closed-off fibrous cord-like structure connected to the bladder; a bladder diverticulum has a hollow urachus at the end connected to the bladder; and a urachal cyst is hollow in the middle with both ends closed.^[1]^

Urachal remnants are occasionally accompanied by umbilical cord cysts and a large umbilical cord, resulting in under-diagnosis or misdiagnosis. Delayed diagnosis causes subsequent complications such as infection, abdominal pain, and malignant transformation. In this report, we describe the findings of a newborn who was referred for a giant umbilical cord cyst, diagnosed as a patent urachus with radiologic images, and surgically treated.

## 2 Case report

**Figure 1:**
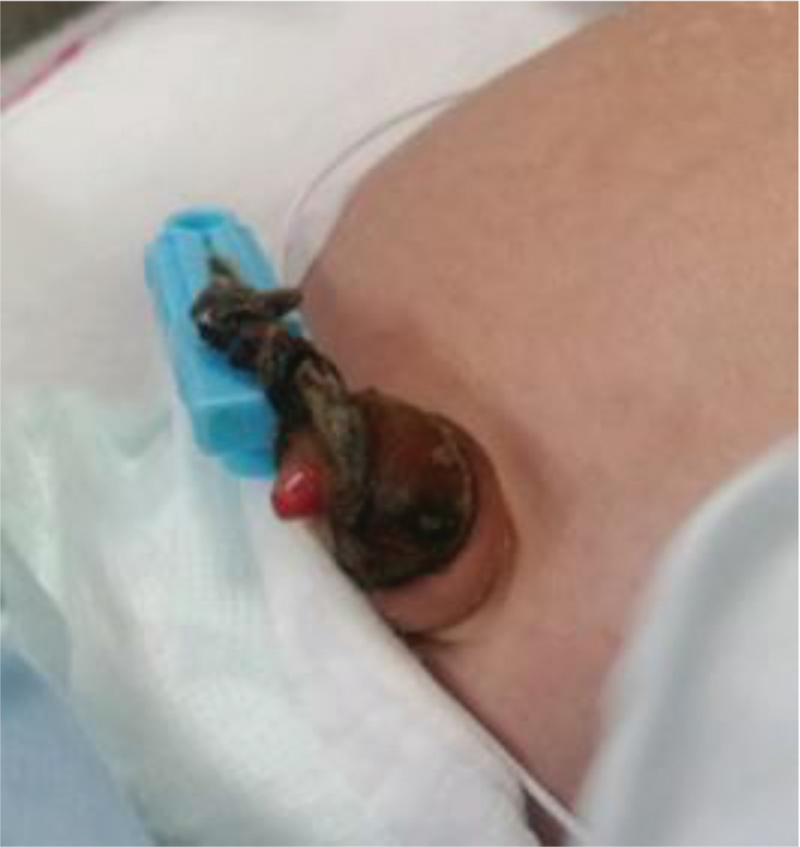
The mummified large umbilical cord with bright red granuloma and pinpoint opening of the fistula.

A male infant weighing 3260 g was born via vaginal delivery without any perinatal problems at a gestational age of 40 weeks. After the infant was born, the umbilical cord was found to be large, and fetal sonographic examination revealed an umbilical cord cyst which prompted a referral to Chungbuk National University hospital's pediatric outpatient clinic on the sixth day of life. Physical examination revealed a large umbilical cord but no other significant findings, such as discharge, and ultrasound examination was scheduled to rule out urachal anomalies. At the 1-week follow-up, the umbilical cord showed a yellowish discharge and granulation with a bright red umbilical polyp and pinpoint opening (Fig. 1). Ultrasound examination revealed a prominent umbilical remnant with suspicious umbilical sinus finding, with no remarkable findings in the kidneys, bowel, liver, or any other intra-abdominal organs. The patient was admitted to the neonatal intensive care unit with the impression of an umbilical sinus.

**Figure 2:**
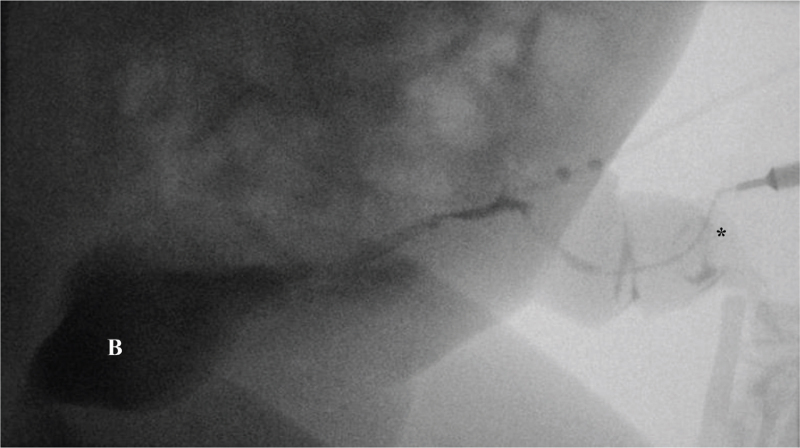
Fistulography reveals a tubular connection between the urinary bladder (B) and the umbilicus (^∗^).

Fistulography was performed through a pinpoint opening of the umbilicus. Contrast examination revealed an enhanced tubular structure within the umbilicus and drainage of the contrast agent into the bladder, confirming the diagnosis of patent urachus (Fig. 2). The patient underwent a planned surgical excision of the urachal remnant with partial cystectomy.

**Figure 3:**
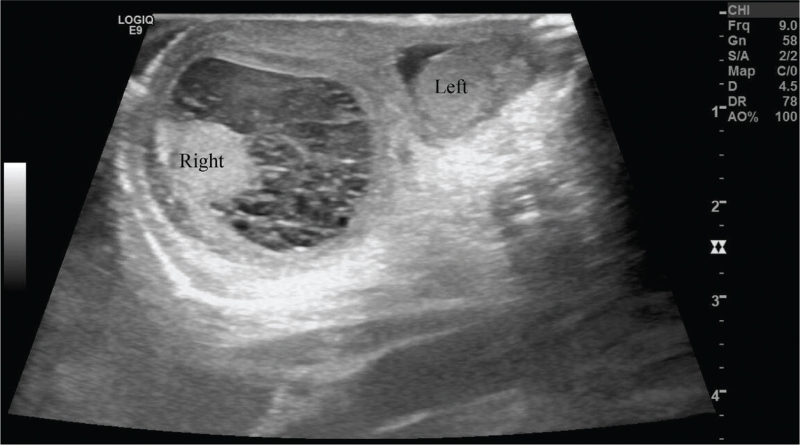
Transverse sonographic image demonstrating the right-sided heterogenous fluid collection with septations suggesting pyocele.

On the second postoperative day, while the umbilical surgical site was clear, the right scrotum was enlarged, and on the next day, the scrotum showed redness as well as hardness and tenderness upon palpation, indicating inflammation. Scrotal ultrasound confirmed orchitis with pyocele of the right scrotum along with innumerous internal septae and a hypervascular right testicle (Fig. 3). A blood test also supported these findings, showing an elevated C-reactive protein level of 1.63 mg/dL (reference >0.3 mg/dL). The initial umbilical swab culture obtained on the day of admission showed colonization of methicillin-resistant *Staphylococcus aureus* and *Escherichia coli*, prompting antibiotic treatment with vancomycin and meropenem. After administration of antibiotics for 10 days, swelling, hardness and redness of the right scrotum subsided and C-reactive protein levels normalized. Voiding cystourethrography (VCUG) was performed before discharge to examine the bladder volume, bladder drainage, and the presence of any structural abnormalities of the bladder. No leakage of the contrast material was seen upon filling the bladder nor were signs of reflux or leakage seen upon voiding. The patient was discharged on the 15th postoperative day and showed no further complications after the surgical removal of the patent urachus and antimicrobial therapy for orchitis and pyocele.

The outpatient postoperative assessment, which was conducted on the 21st postoperative day, revealed no remarkable complications in the umbilical surgical site, in addition to the gross physical examination and ultrasound findings of the scrotum and spermatic cord. The patient also showed adequate growth after being discharged from the hospital.

## 3 Discussion

A patent urachus was diagnosed in a male newborn with a large umbilical cord, and an umbilical cord cyst was diagnosed based on radiological images. The patient underwent planned surgical repair and was treated with antibiotics for concurrent orchitis and pyocele. The patient presented with no further complications and was healthy on discharge.

Umbilical remnants originate from partial or total failure of obliterations between the fetal bladder and the allantois. A patent urachus indicates complete communication between the bladder and the umbilicus. It is often suspected prenatally in cases showing ultrasound images of umbilical cord cysts with or without bladder problems such as megacystis and bladder eversion caused by reflux of urine into the umbilical cord resulting in an allantoic cyst during fetal life.^[1,4]^ In this case, the patient was referred for a giant umbilical cord and a suspected umbilical cord cyst on prenatal ultrasound. However, postnatal ultrasonography and fistulogram obtained via the opening of the umbilicus revealed a patent urachus. After birth, abnormal physical findings, including persistent umbilical discharge or umbilical polyps, should raise suspicion for urachal remnants, which can be evaluated by imaging examinations such as postnatal ultrasound, VCUG, fistulography, or computed tomography to discriminate urachal remnants from benign lesions. Concurrent genitourinary malformations should be investigated with imaging studies such as intravenous pyelography and VCUG.^[2]^ We observed a negative result in VCUG in the present case.

There is insufficient consensus regarding the choice between conservative therapy and surgical resection in the management of urachal remnants because of their rare occurrence. Cuda et al. reported successful spontaneous closure of a patent urachus by conservative management with urethral catheterization.^[4]^ Nevertheless, surgical excision is generally recommended for urachal remnants because of the possibility of recurrent infections and malignant transformation.^[2,5,6]^ Conservative treatments such as antibiotics and urethral catheterization are associated with a risk of potential failure and require repeated imaging studies to identify the spontaneous resolution of urachal remnants. On the other hand, surgical repair is preferred in patients with symptomatic urachal remnants. A patent urachus is usually symptomatic; in this case, our patient showed yellowish umbilical discharge and underwent a planned operation for urachal remnant resection.

Postoperative complications are uncommon in patients with urachal remnant. Previous studies reported that the incidence of wound infections, bladder leakage, and reoperation was 15%.^[6,7]^ In our case, the infant showed a unilateral erythematous, swollen scrotum with tenderness, indicating inflammation or infection, on the day after the surgical repair, and was diagnosed with orchitis and pyocele based on scrotal ultrasound findings. The postoperative wound was clear.

The cause of pyocele is either idiopathic or infectious, with the condition manifesting as an intraperitoneal infection spreading through a patent processus vaginalis testis or as a primary infection of the testis, epididymis, or testicular appendage.^[8,9]^ In the present case, the pyocele was considered an adverse outcome of fistulography or a preoperative intra-abdominal infection from patent urachus or postoperative inflammation. Pyocele is a rare urological emergency condition that requires early diagnosis and intervention to prevent the development of sepsis and testicular loss. We performed an immediate ultrasound to diagnose pyocele and consulted a urologist for management. Although broad-spectrum antibiotic treatment with surgical drainage is known to be effective for the treatment of pyocele, in this case, pyocele was resolved by prompt administration of antibiotics without surgical exploration. We did not determine the cause of pyocele and the organism of the infection without drainage of the fluid collection. However, the patient was successfully treated with antibiotics without testicular loss and showed no further complications.

## 4 Conclusion

A patent urachus is a rare urachal remnant. Careful evaluation should be performed in neonates with umbilical cord cysts or large umbilical cords to distinguish between coexisting urachal anomalies. Herein, we report a case involving successful treatment of a newborn with patent urachus concurrent with pyocele and orchitis, which may have been a possible complication of patent urachus.

## Author contributions

SJK and EI performed the study, participated in the data collection, and drafted the manuscript. YJK, JHL, and JWP designed the study. SAY substantially revised the manuscript. All the authors have read and approved the final version of the manuscript.

**Conceptualization:** Jin-Woo Park, Shin Ae Yoon, Yoo-Jin Kim.

**Data curation:** Erika Imura, Ji Hyuk Lee, Shin Ae Yoon, Susie Kang.

**Investigation:** Yoo-Jin Kim.

**Supervision:** Ji Hyuk Lee, Jin-Woo Park, Shin Ae Yoon.

**Writing – original draft:** Erika Imura, Susie Kang.

**Writing – review & editing:** Shin Ae Yoon.
